# Two-dimensional distributed-phase-reference protocol for quantum key distribution

**DOI:** 10.1038/srep36756

**Published:** 2016-12-22

**Authors:** Davide Bacco, Jesper Bjerge Christensen, Mario A. Usuga Castaneda, Yunhong Ding, Søren Forchhammer, Karsten Rottwitt, Leif Katsuo Oxenløwe

**Affiliations:** 1 Technical University of Denmark, Department of Photonics Engineering, 2800 Kgs. Lyngby, Denmark

## Abstract

Quantum key distribution (QKD) and quantum communication enable the secure exchange of information between remote parties. Currently, the distributed-phase-reference (DPR) protocols, which are based on weak coherent pulses, are among the most practical solutions for long-range QKD. During the last 10 years, long-distance fiber-based DPR systems have been successfully demonstrated, although fundamental obstacles such as intrinsic channel losses limit their performance. Here, we introduce the first two-dimensional DPR-QKD protocol in which information is encoded in the time and phase of weak coherent pulses. The ability of extracting two bits of information per detection event, enables a higher secret key rate in specific realistic network scenarios. Moreover, despite the use of more dimensions, the proposed protocol remains simple, practical, and fully integrable.

Sharing sensitive information has always been a great challenge within our society. In particular, QKD, first introduced by Bennett and Brassard, provides a unique procedure for exchanging a private key, based on the laws of quantum mechanics^1^. During the last decade, the effort from the scientific community has been focused on an enhancement of the quantum communication performances in terms of key rate, transmission distance and security aspects^2^,^3^,^4^,^5^,^6^,^7^,^8^,^9^. In later years this technology has matured enormously, but the lack of compact, efficient, inexpensive, and reliable systems, has restricted wide spreading of practical QKD systems.

The basic idea behind QKD systems, in the case of “prepare and measure” schemes, is based on quantum states prepared by Alice (the transmitter) and sent through a quantum channel towards Bob (the receiver). Depending on the quantum measurement, Bob can deduce which state was prepared by Alice. This way, after error reconciliation and privacy amplification methods established in a classical channel, the two users share an identical bit sequence.

Ideally, QKD systems are secure with no chance for an eavesdropper to extract information on the key. However, in real implementations of the systems, due to the losses and imperfections of devices, the secret key rate defines a bound on how much information can be assumed secure^10^,^11^,^12^.

We here propose a new QKD protocol, which we refer to by the name: Differential phase time shifting (DPTS). In its essence, the protocol utilizes two degrees of freedom — time and phase — to encode information in a quaternary alphabet, i.e. {0, 1, 2, 3}^13^. The DPTS belongs to the family of distributed phase-reference (DPR) protocols, which rather than using the principle of random basis-choices between different mutually unbiased bases, encodes information in adjacent weak coherent pulses^6^,^10^,^14^,^15^,^16^,^17^,^18^. We study the performance of the DPTS protocol using infinite-key analysis in the case of collective attacks, and further show that the protocol holds great potential in intracity network scenarios.

## Results

### Principle of DPTS

**Figure 1:**
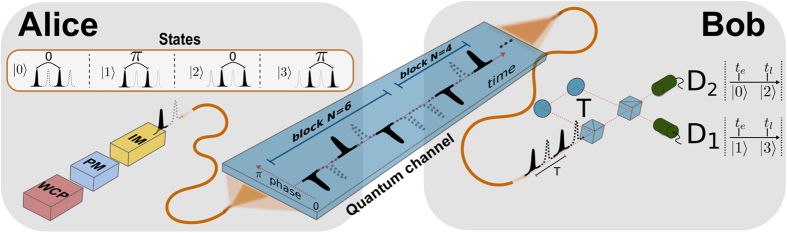
Basic scheme of the DPTS protocol exploiting phase and time domain. A train of weak coherent pulses (WCP) is emitted by a laser of repetition rate *ν* (2/*T*), and attenuated to the single photon level. A phase modulator (PM) encodes the first key bit in second-neighbor pulses with a period of *T* by choosing randomly either 0 or *π*. An intensity modulator (IM) is used to choose the position of the pulses to encode the second key bit. The number of pulses *N*, where the intensity modulator uses the same time instances, is defined as a *block*. In this way, Alice prepares a sequence of different states: |0〉, |1〉, |2〉, |3〉. Using a delay line interferometer with a delay of *T* between arms, Bob can simultaneously measure the phase and pulse position.

As in most practical implementations of QKD, the DPTS protocol, which is sketched in Fig. 1, uses a source of weak coherent pulses to establish a key of random numbers between two authenticated parties, Alice and Bob. To initiate the key distribution process, Alice randomly encodes information in the train of pulses in two dimensions, time and phase. *The time encoding* is performed using an intensity modulator (IM) as in the coherent-one way (COW) protocol^15^. For every pair of pulses (we refer to such a pair as a *sub*-*block*), one pulse is transmitted with mean photon number *μ* < 1 (|*α*〉), and one is blocked completely (|vac〉). Hence, within each sub-block, information is carried by the time-of-arrival of a non-empty pulse^15^,^19^. *The phase encoding* is performed using a phase modulator (PM), where a random phase between sub-blocks is either {0, *π*}. By combining the effect of the IM and the PM, Alice prepares states from the quaternary alphabet:


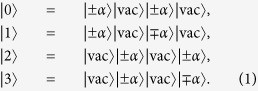


Bob may distinguish unambiguously between these states by employing an unbalanced interferometer which interferes pulses in adjacent sub-blocks separated by *T* = 2/*ν*, where *ν* is the laser repetition rate. Depending on the time of arrival (*t*_*e*_ or *t*_*l*_ in Fig. 1) and on which detector fired (*D*_1_ or *D*_2_), Bob can decide which of the four states was prepared. We would like to point out that, due to the used interferometer delay, no interference occurs in the case of a transition sequence, such as |±*α*〉 |vac〉 |vac〉 |±*α*〉.

It is important to note that, analogous to the differential phase shift (DPS) protocol, each sub-block may participate in defining up to two states^14^. For instance, the sequence: |*α*〉 |vac〉 |−*α*〉 |vac〉 |*α*〉 |vac〉 |vac〉 |*α*〉 |vac〉 |−*α*〉 encodes the states: |1〉 |1〉 − |3〉. Here, the ‘−’ indicates a change of the temporal sequence over the sub-block separation, in which case Bob is not able to interfere the non-empty pulses in his interferometer (for a detailed example, see Supplementary information).

To minimize the number of transition sequences, Alice and Bob may benefit from repeating the temporal encoding over long pulse intervals (i.e. only preparing |0〉 and |1〉, or |2〉 and |3〉). However, doing so permits a potential eavesdropper, Eve, to gain partial information on a given state by measuring the time-of-arrival of pulses in adjacent sub-blocks. This effectively means that the time-of-arrival information is more vulnerable to eavesdropping. To counteract this potential attack, Alice introduces the concept of *blocks*. Each block consists of *N* pulses (counting both empty and non-empty), within which the temporal sequence is repeated independently from the previous block (the sequences |0〉 |1〉 |1〉 and |3〉 |2〉 are examples of blocks with *N* = 8 and *N* = 6). The value of *N* is for each block chosen randomly from a uniform distribution: *N* ∈ {4, 6, …, *N*_*max*_}. In contrast, if the value of *N* was fixed at e.g. *N* = 6, then Eve would know exactly for which sequences of pulses the temporal encoding was repeated. The modification of random block lengths, means that both Bob and Eve are essentially unaware of the positions of the block separations. Whereas this is of no importance to Bob (see section ‘Protocol definition’), it is fundamental to Eve.

The security of DPTS relies on the same principle as other DPR protocols: the coherence between non-empty pulses^20^,^21^. In fact, the DPS aspect of the DPTS protocol makes it very robust against attacks such as the intercept-resend attack and the photon-number splitting attack^21^,^22^. Eve can not perform a measurement on any finite number of states without at some point breaking coherence between successive pulses. This is specifically true for the DPTS protocol as Eve is not able to predict the positions of the transition sequences. However, since coherence is distributed across sub-block separations whereas the temporal information lies within sub-blocks, a sophisticated Eve can address each sub-block separately trying to just learn the time-of-arrival information (i.e. is a state |0〉, |1〉 or is it |2〉, |3〉). Doing so, she only breaks coherence *within* sub-blocks, and thus Bob, who only checks coherence *across* sub-blocks, is not able to reveal her presence. To counter this attack, Alice introduces decoy sequences with probability 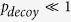, in which blocks consist of *N* non-empty pulses^20^. Interestingly, this decoy is just a DPS sequence in which the phase encoding is carried between every second pulse (as measured by Bob). Consequently, if Eve probes one or more sub-blocks containing two non-empty pulses, she inevitably disturbs the phase relation between these pulses^11^. As a result, there are cases where Eve introduces phase errors into the communication.

### Protocol definition

We now describe in detail how Alice and Bob establish a common key using the DPTS protocol:

- Alice prepares states for transmission in the quantum channel using her phase- and intensity modulators. We assume that Alice chooses equally and randomly between the four different states {0, 1, 2, 3}. The temporal sequence is repeated within each block of random length, *N* ∈ {4, 6, …, *N*_*max*_}, whereas the phase difference between each sub-block is randomly chosen to either 0 or *π*.
- Once Bob has received a photon in one of the two detectors, he reveals over a public classical channel the sub-time (the number of the sub-block) instances of his recorded detection events.
- Alice reports back by telling which of the events corresponded to an overlap between adjacent blocks with opposite temporal sequence (a block separation was present in that instance). Bob must discard these events.
- For each of the remaining detection events, Alice and Bob establish two bits of information for their key: Alice easily figures out the detection time from her sent temporal sequence, and infers from her phase encoding which detector clicked at Bob’s side.
- After estimating the quantum bit error rate (QBER), Alice and Bob perform standard error reconciliation and privacy amplification^23^,^24^,^25^. At the end of the process Alice and Bob share a secure identical key.

### Secret key rate

To further describe the proposed protocol, let us consider the maximum extractable secret key rate *R*_*sk*_^11^. For the DPTS protocol this quantity reads


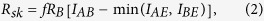


where *R*_*B*_ = *R* + 4*p*_*d*_(1 − *R*) is the total detection rate with *R* = [1 − exp(−*μtη*_*d*_)]/2, *μ* is the mean photon number of non-empty pulses, *t* represents the quantum channel transmission coefficient, *η*_*d*_ is the (common) detector efficiency, and *p*_*d*_ is the dark count probability. The pre-factor *f* = (1 − *p*_*decoy*_) (〈*N*〉 − 1)/〈*N*〉, where 〈*N*〉 = (*N*_*max*_ + 4)/2 is the average block length, takes into account the fraction of Bob’s detection events that is assigned to the key string. The unused fraction 1/〈*N*〉 is due to detections associated with adjacent sub-blocks of different temporal sequences. In these cases, the clicks are randomly distributed between the two detectors, and so the instances are discarded.

The mutual information between Alice and Bob, is expressed in terms of the Shannon entropy as *I*_*AB*_ = *H*(*A*) − *H*(*A*|*B*)^26^. Alice has a total of four different states to choose from, and by assuming that she prepares each state with equal probability, one finds 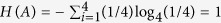. Note that we, for convenience, measure information using a base-4 logarithm rather than the common base 2 [in units of bits one acquires *H*(*A*) = 2]. Furthermore, the conditional entropy *H*(*A*|*B*) is expressed as


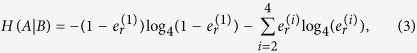


where the four error probabilities satisfying 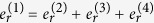 are given as


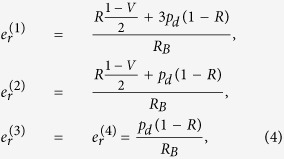


where 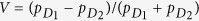 represents the visibility of the interferometer used by Bob and 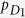 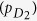 represents the probability of detection in detector *D*_1_ (*D*_2_). Note that, in the definition of the error probabilities, the visibility appears in only two of the four terms, since an interferometer error does not alter the time of arrival. Thus, since the time-of-arrival information remains correct, the DPTS protocol suffers less from interferometer imperfections in comparison with the DPS protocol which solely relies on relative phase measurements. On the other hand, the higher dimensionality of the DPTS protocol renders it more vulnerable to detector dark counts: each dark-count occurrence results in two random bits rather than one. This effectively makes the DPTS protocol less useful at longer communication distances where the dark count rate becomes comparable with the signal rate.

In order to evaluate the achievable secret key rate for Alice and Bob, we next introduce an upper bound on the information that a potential eavesdropper might obtain by performing the most basic attack; the beam-splitting attack. In the family of collective attacks, Eve is assumed to be able to interact with the same strategy on a predefined number of pulses. She can store the photons and try to extract the largest possible information after Alice and Bob has performed post-processing. A complete analysis would concentrate on *I*_*BE*_ since Eve is clueless about detection events resulting from imperfections at Bob’s side (see equation (2)). However, as a first attempt to estimate her information, we restrict ourselves to the more simple analysis of *I*_*AE*_.

### Security analysis

This section presents an analysis of security based on the collective beam-splitting attack (BSA) and follows the method used in ref. 27 for the DPS and COW protocols. In the BSA, Eve replaces the quantum channel connecting Alice and Bob by a lossless line. Using a beam-splitter to simulate the losses of the quantum channel, Eve acquires 1 − *t* of the signal without disturbing the state sent by Alice. Thus, the BSA belongs to the family of zero-error attacks, and is therefore undetectable by Alice and Bob^28^. The states prepared by Alice consist of sequences 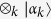 with *α*_*k*_ ∈ {+*α*, vac, −*α*}, so by performing the BSA, Eve receives states of the form 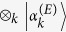, where 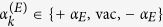 with 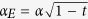.

At this point we assume that Eve stores the states in her quantum memory for measurement after Bob reveals his detection events. Indeed, for such a collective attack, the maximum information she may extract is given by the Holevo quantity (which must be maximized with respect to the strategies available to Eve, though here we only consider the BSA)^11^,^29^


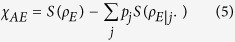


Here, *S*(*ρ*) = −Tr {*ρ* log_4_ (*ρ*)} is the von Neumann entropy, 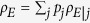 is a density operator with *p*_*j*_ being the probability of Alice preparing the four states *j* ∈ {0, 1, 2, 3}, and *ρ*_*E*|*j*_ being Eve’s state conditioned on preparation of state *j*.

As mentioned earlier, we consider only the balanced situation where Alice prepares each state with a probability *p*_*j*_ = 1/4. In the current protocol each value in the quaternary alphabet is encoded in four consecutive pulses. It follows that Eve’s states conditioned on Alice’s preparation are


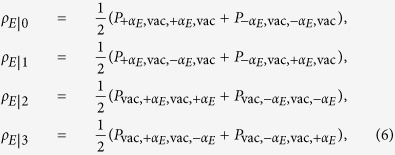


where *P*_*x*_ is the projection operator. To calculate the maximum accessible information for Eve, it is helpful to define 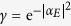. By this convention the overlaps between states can be written as 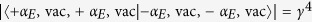, and |〈*j*|*k*〉| = *γ*^2^ for *j* ≠ *k*, where *j, k* ∈ {0, 1, 2, 3}. By writing *ρ*_*E*_ and *ρ*_*E*|*j*_ in their respective eigenbasis, the von Neumann entropy takes the simple form 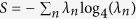, where *λ*_*n*_ are the eigenvalues. The resulting Holevo quantity is


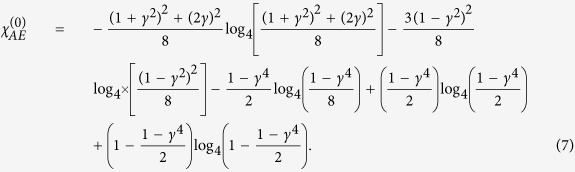


and presents an upper bound on the information Eve can obtain by trying to distinguish between the four different states after Bob announces a detection event.

In the cases where Eve fails to get a conclusive measurement, she may instead try to establish partial information about the state Alice and Bob agreed upon. She can do this by trying to measure the temporal position (i.e. is a state |0〉, |1〉 or |2〉, |3〉) of the pulse in a sub-block adjacent to the sub-block corresponding to Bob’s detection. In general for the considered block lengths, the probability of this measurement to be correct (if conclusive) exceeds 1/2 (for details, see Supplementary information), and thereby effectively provides Eve with information on the state. Since this additional attack by Eve is conditioned on her *not* getting a conclusive result in the primary measurement, the corrected Holevo quantity becomes


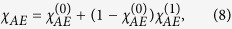


where 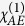 is derived and given in the Supplementary information. Note however, that Eve is essentially ignorant about the position of block separations. Therefore, making conclusions based on this secondary attack will result in errors for Eve, effectively reducing the gained information.

### Numerical results

**Figure 2:**
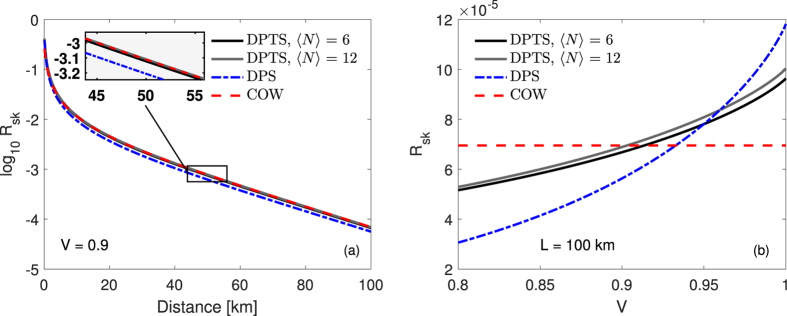
Secret key rate measured in bits per pulse. Performance versus (**a**) distance in the case of fixed visibility, *V* = 0.9, and (**b**) visibility at a channel length of *L* = 100 km. For each of the three protocols, an optimization was performed with respect to the mean photon number *μ* (see Supplementary Fig. S2). Parameters: *η*_*d*_ = 0.1, *p*_*d*_ = 2 × 10^−8^, *α*_*loss*_ = 0.2 dB/km, and *p*_*decoy*_ = 0.02 for COW and DPTS.

Combining the results of the previous sections (equations (2, 3, 4, 5, 6, 7, 8)) the secret key rate for DPTS reads 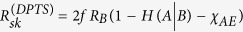, where the factor of two stems from the conversion from a quaternary to binary alphabet. This expression enables us to plot a first upper bound on the secret key rate under the assumption of collective attacks. Specifically, Fig. 2 shows *R*_*sk*_ versus communication distance at the optimized values of the mean photon number *μ*. To assess the performance of the DPTS protocol, we have included plots for both COW and DPS. The secret key rate for COW and DPS are obtained by: 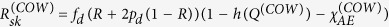 and 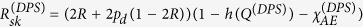, where *R* is defined below equation (2), *Q*^(*COW*)^ and *Q*^(*DPS*)^ are the quantum bit error rate for COW and DPS respectively, *f*_*d*_ represents the decoy state probability, *h*(*Q*) is the binary entropy and the Holevo bounds 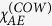 and 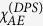 are defined in Branciard *et al*.^27^. These equations are derived under the same assumptions as made for the DPTS protocol to allow for a fair comparison. As a result, the COW protocol does not exhibit any visibility dependence (see Fig. 2(b)).

In comparison, the DPTS protocol has a similar performance as the other protocols under the realistic condition of non-ideal visibilities (as example we have used *V* = 0.9). Noteworthy, the DPTS protocol displays a less critical dependence on the visibility when compared to the DPS protocol.

**Figure 3:**
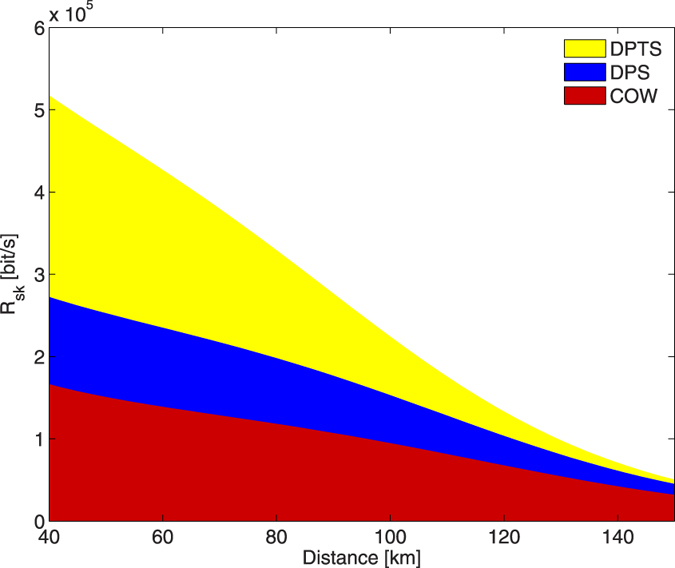
Secret key rate in real case scenario. Different secret key rates achievable in a medium-length link scenario, where the detector dead times play an important role. We use mean photon numbers for the different protocols of *μ*_*DPTS*_ = 0.23, *μ*_*DPS*_ = 0.19, and *μ*_*COW*_ = 0.52, at repetition rate *ν* = 2 GHz, and average block length of 〈*N*〉 = 6. The detectors are specified by dark-count probability *p*_*d*_ = 2 × 10^−8^, a dead time of *t*_*d*_ = 2 *μ*s, and efficiency *η*_*d*_ = 0.1. We assume *V* = 0.98, and a decoy-sequence probability of *p*_*decoy*_ = 0.02 for COW and DPTS.

In a more realistic situation, the comparison of the protocols must take into account the detector dead times. For example, considering the case of commercial InGaAs infrared single-photon detectors (the most used in fiber links and the most promising thanks to the non-cryogenic requirement), they generally exhibit a dead time in excess of 1 *μ*s^30^,^31^. Thus, in any scenario where the detector dead time significantly influences the key generation rate, the ability to extract two bits of information per detection event grants the DPTS protocol an advantage. To illustrate this effect, Fig. 3 shows an example of the secret key rate in bits s^−1^, after inclusion of the dead-time dependency.

## Discussion

The main figure of merit in a QKD system is the achievable secret key rate. Therefore, to assess the performance of DPTS, Fig. 2 displays this quantity for DPTS in comparison with the standard COW and DPS protocols. The comparison shows very similar behavior of the three DPR protocols. Considering more specifically the case of DPTS, the final key rate is influenced by the length of the blocks *N* prepared by Alice. Even though a higher value of *N* allows an increased sifted key rate, it is necessary to consider a trade-off between the length of blocks and the information leakage to Eve. In the case of long-distance links (in excess of 100 km), the behavior of the three protocols is maintained, but as the DPTS protocol is more severely influenced by dark count events, it is generally limited to shorter distances. On the other hand, as seen from Fig. 2(b), the DPTS protocol is less dependent on the interferometer visibility. This fact permits the proposed protocol to achieve a more stable secret key generation rate in comparison with the DPS protocol.

In implementing a QKD protocol, it is necessary to consider the limitations set by the optical and electronic devices^32^,^33^,^34^. An important example is the single-photon detector dead time *t*_*d*_, which sets an upper limit on the key generation rate. This parameter is important in a short- or medium-length link scenario, where the average wait time between detection events is of the same order of magnitude as *t*_*d*_ (which is typically on the order of microseconds). In Fig. 3, it is shown that DPTS may achieve a significant increase in the secure key rate at distances where the detector dead time is a limiting factor. This potential arises due to the ability of the DPTS protocol to extract two bits of information per detection event.

The use of multiple degrees of freedom in transmission of information, intuitively increases the complexity of the scheme in comparison with protocols dealing with each individual degree of freedom. Despite DPTS not being an exception to this rule of thumb, the complexity overhead in comparison to DPS or COW is not crucial. On the other hand, DPTS does exhibit two significant practical advantages. Firstly, the COW protocol requires a monitoring line to check for the presence of an eavesdropper. However, such a monitoring line is unnecessary for DPTS, as an interferometer is directly used in the data line, and hence implements the necessary coherence check. Thus, the decrease in rate related to monitoring of the data line in COW, is not a limitation for DPTS. Secondly, the stability of the interferometer over time, is a considerable challenge in implementations of the DPS protocol in non-stable environments. The performance of the DPTS protocol is inherently more resilient against fluctuating interferometer visibilities, because the temporal bit remains unaffected by such inefficiencies. This entails, that DPTS might be better suited in cases where it is difficult to maintain the interferometer visibility above a certain required operation threshold.

Finally, DPTS can potentially play an important role in QKD networks spanning from metropolitan to intercity distances^35^,^36^,^37^,^38^,^39^. Interestingly, the required measurement apparatus is identical to the one used in DPS, and in fact, the receiver does not need to know *a priori* whether the signals arise from a DPS or a DPTS encoding. This compatibility suggests that a versatile network encompassing the use of both the DPS and DPTS protocols is feasible.

In conclusion, we have proposed a novel kind of distributed-phase-reference protocol for quantum key distribution. Utilizing both the time- and phase degrees of freedom, this protocol provides a significant step towards realization of fast, reliable, and practical quantum communication. Future directions include a finite-key analysis and a real-time field implementation.

## Additional Information

**How to cite this article**: Bacco, D. *et al*. Two-dimensional distributed-phase-reference protocol for quantum key distribution. *Sci. Rep.* **6**, 36756; doi: 10.1038/srep36756 (2016).

**Publisher's note:** Springer Nature remains neutral with regard to jurisdictional claims in published maps and institutional affiliations.

## Supplementary Material

Supplementary Information
