# Supplementary material for: Two-dimensional distributed-phase-reference protocol for quantum key distribution
Source: Sci Rep. 2016 Dec 22;6:36756. doi: 10.1038/srep36756 (PMC5177871; doi:10.1038/srep36756)
Supplement: Supplementary Information [file srep36756-s1.pdf]

Supplementary Information: Two-dimensional distributed-phase-reference protocol for quantum key distribution

Davide Bacco[1*+], Jesper Bjerge Christensen[1+], Mario A. Usuga Castaneda[1], Yunhong Ding[1], Søren Forchhammer[1], Karsten Rottwitt[1], and Leif Katsuo Oxenløwe[1]

[1]Technical University of Denmark, Department of Photonics Engineering, 2800 Kgs. Lyngby, Denmark.
[*]dabac@fotonik.dtu.dk
[+]these authors contributed equally to this work

DPTS example

In this section we describe a concrete example of the DPTS protocol. In particular, Figure S. 1 shows the protocol structure in all the consecutive processes. A sequence of quantum states is generated by a random number generator ($|1\rangle, |1\rangle, |0\rangle, \ldots$). Alice uses an intensity modulator and phase modulator to encode the quantum states into the train of weak coherent pulses. The red vertical lines indicate block separations, for which there is a 50% probability of having a change in the pulse-position encoding (a transition sequence, $-$). In the case of a transition sequence, a measurement by Bob does not result in any key transfer. This is exemplified by subslots #5 and #11, which thereby indicate a useless measurement. Subslot #7, even though it corresponds to a block separation, can be used for the key extraction. After the physical transmission of the quantum states, Bob reports back, through the classical channel, the numbers of the active subslots (where his detectors have fired). At this point, Alice will select only the corresponding subslots and tells Bob to discard those of his measurements which corresponded to transition sequences. Finally, Alice and Bob, after error correction and privacy amplification, share a quantum key that can be used for encryption and decryption of the plain text.

Eve's additional attack

We here explore an additional (or secondary) attack option which is available to Eve when performing the beam-splitting attack (BSA). The possibility of this additional attack, arises as Alice repeats the temporal sequence (i.e. non-empty, empty or empty, non-empty) within each block of length N. To clarify, assume that Bob has a detection event in a certain time slot. Eve, wanting to know which state Alice prepared for Bob, extracts the corresponding 4-pulse state from her quantum memory, and tries to determine whether it was $|0\rangle, |1\rangle, |2\rangle$ or $|3\rangle$ (See Main Text, Security analysis). Often, Eve has an inconclusive measurement and the state of the 4-pulse system is destroyed. However, in these cases, she can extract an adjacent 2-pulse state from her quantum memory, and try to learn its temporal encoding (i.e. is it $|0\rangle, |1\rangle$ or $|2\rangle, |3\rangle$), which is worth 1 bit of information. Unfortunately for Eve, this bit will not always be correct: In some cases she extracts a 2-pulse state belonging to an adjacent block of the opposite temporal encoding. And, essentially for the protocol, she does not know when this is the case due to the randomized block length.

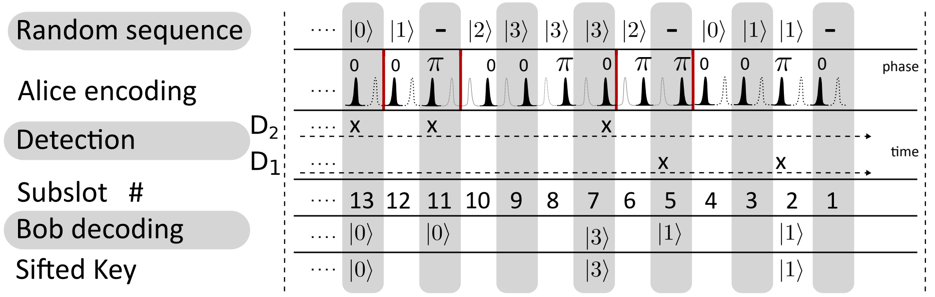

Figure S1. *Practical example of DPTS protocol.* The red lines indicate a block separation. '$-$' represents a transition sequence between blocks. Such a transition sequence is useful for key extraction with a probability of 50%.

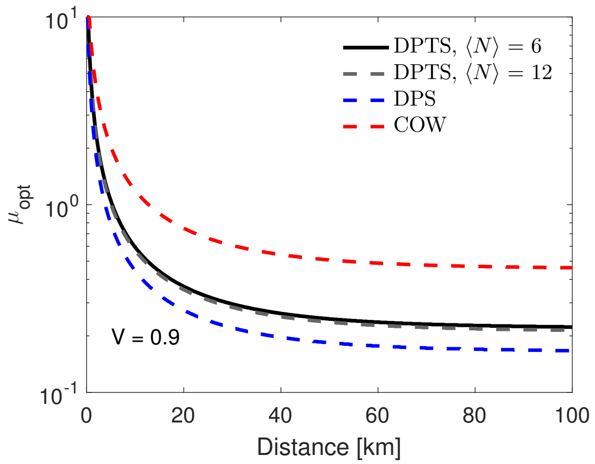

Figure S2. *Optimal μ versus distance.* Mean number photon per non-empty pulse versus distance for the DPTS ($\langle N\rangle = 6$ and $\langle N\rangle = 12$), DPS and COW protocols. The corresponding secret key rates are shown in Fig. 2.

The probability of a correct bit for Eve p_E, depends on the average block size $\langle N\rangle$, and can be found to satisfy (assuming a negligible fraction of decoy sequences)

$$p_E = \frac{\langle N\rangle - 2}{\langle N\rangle - 1}, \qquad \langle N\rangle > 4, \tag{1}$$

which tends towards unity for $\langle N\rangle \gg 4$ as intuitively expected. Since the nature of the errors are identical to those of a binary symmetric channel (BSC), we can explicitly express the correction term in equation (8) as

$$\begin{aligned}\chi_{AE}^{(1)} = \frac{1}{2}\left[1 - h(p_E)\right]\Bigg[&-\left(\frac{1+3\gamma^2}{4}\right)\log_4\left(\frac{1+3\gamma^2}{4}\right) - 3\left(\frac{1-\gamma^2}{4}\right)\log_4\left(\frac{1-\gamma^2}{4}\right)\\ &+\left(\frac{1-\gamma^2}{2}\right)\log_4\left(\frac{1-\gamma^2}{2}\right) + \left(1-\frac{1-\gamma^2}{2}\right)\log_4\left(1-\frac{1-\gamma^2}{2}\right)\Bigg].\end{aligned} \tag{2}$$

The pre-factor of $1/2$ enters since this attack only gives half of the state information, the factor $1 - h(p_E)$ is the BSC capacity, and finally the terms in the last square bracket results from analysing how well Eve can discriminate unambiguously between the two different temporal sequences. Note that this is not identical to the expression for the coherent-one-way protocol, since Eve's conditioned states in our case are: $\rho_{E|0} = (P_{+\alpha_E,\text{vac}} + P_{-\alpha_E,\text{vac}})/2$ and $\rho_{E|1} = (P_{\text{vac},+\alpha_E} + P_{\text{vac},-\alpha_E})/2$.

The corrected Holevo bound presented in this section only takes into account a single additional measurement performed by Eve. In principle, this measurement may be inconclusive in which case she can extract a new 2-pulse state and perform a new measurement. Thus, a more accurate analysis does exist, but is considered outside the scope of this paper as it is not expected to have a crucial impact on the bound for $\langle N\rangle \leq 8$.

Mean photon number parametrization

The secret key rate R_{sk} in equation (2) indicates that one should always try to optimize $I_{AB} - \min(I_{AE}, I_{AB})$ with respect to the free variables available. For a given transmission link, an obvious parameter to optimize is the mean photon number per pulse μ. In general, Bob's detection rate increases with μ, but so does Eve's probability of measuring the corresponding state. Thus, for a specific setup (QKD protocol, transmission channel, interferometer, detectors, etc.), it is expected that an optimal value, μ_{opt}, exists. As an example, Supplementary Fig. 2 shows the behavior of μ_{opt} versus transmission distance. These values were, for each transmission distance, obtained by numerically finding the value μ_{opt}, which optimized R_{sk} (which is then shown in Fig. 2). As the DPTS protocol forces a potential eavesdropper to distinguish both between states $|+\alpha\rangle, |-\alpha\rangle$ (as in DPS) and $|\pm\alpha\rangle, |\text{vac}\rangle$ (as in COW) it is perhaps not surprising that the optimal value μ_{opt} for the DPTS protocol lies somewhere in between the corresponding optimal values for DPS and COW.
